# Simple Shading Correction Method for Brightfield Whole Slide Imaging

**DOI:** 10.3390/s20113084

**Published:** 2020-05-29

**Authors:** Yoon-Oh Tak, Anjin Park, Janghoon Choi, Jonghyun Eom, Hyuk-Sang Kwon, Joo Beom Eom

**Affiliations:** 1 Gwangju Institute of Science and Technology, the School of Mechanical Engineering, Gwangju 61005, Korea; takuno7@gist.ac.kr (Y.-O.T.); janguri@gist.ac.kr (J.C.); hyuksang@gist.ac.kr (H.-S.K.); 2 Korea Photonics Technology Institute, Intelligent Photonic Sensor Research Center, Gwangju 61007, Korea; anjin.park@kopti.re.kr (A.P.); jheom@kopti.re.kr (J.E.); 3 College of Medicine, Dankook University, Cheonan-si 31116, Korea

**Keywords:** biomedical imaging, biomedical image processing, whole slide imaging, brightfield microscopy, shading correction

## Abstract

Whole slide imaging (WSI) refers to the process of creating a high-resolution digital image of a whole slide. Since digital images are typically produced by stitching image sequences acquired from different fields of view, the visual quality of the images can be degraded owing to shading distortion, which produces black plaid patterns on the images. A shading correction method for brightfield WSI is presented, which is simple but robust not only against typical image artifacts caused by specks of dust and bubbles, but also against fixed-pattern noise, or spatial variations in pixel values under uniform illumination. The proposed method comprises primarily of two steps. The first step constructs candidates of a shading distortion model from a stack of input image sequences. The second step selects the optimal model from the candidates. The proposed method was compared experimentally with two previous state-of-the-art methods, regularized energy minimization (CIDRE) and background and shading correction (BaSiC) and showed better correction scores, as smooth operations and constraints were not imposed when estimating the shading distortion. The correction scores, averaged over 40 image collections, were as follows: proposed method, 0.39 ± 0.099; CIDRE method, 0.67 ± 0.047; BaSiC method, 0.55 ± 0.038. Based on the quantitative evaluations, we can confirm that the proposed method can correct not only shading distortion, but also fixed-pattern noise, compared with the two previous state-of-the-art methods.

## 1. Introduction

Whole slide imaging (WSI), which refers to scanning a whole slide and creating a single high-resolution digital image, is a relatively new technique that solves the narrow field of view (FOV) problem of traditional optical microscopes [1,2,3]. Since the introduction of the automated WSI device in 1999 [4], the WSI devices can acquire faster and more accurate high-quality digital images of the whole slide due to its recent advances in hardware and software technologies [1,5]. These advances have made it possible for pathologists to convert from viewing slides from a microscope to a computer monitor that displays high-resolution whole-slide images [3]. This transfer extends the field of applications of WSI devices to both diagnostic and histopathologic education and proficiency tests [6,7,8,9].

Digital slide images are typically generated by stitching image sequences acquired from different FOVs of a sample into a single whole-slide image. However, shading distortion, caused by uneven illumination [10], can produce black plaid patterns when stitching image sequences acquired to cover the whole slide, which degrades the visual quality of the digital images [11,12,13]. Moreover, the black plaid patterns on the images adversely affect subsequent image analysis, such as segmentation and quantification [14].

Previous shading correction methods can be classified into two categories: prospective and retrospective [14,15]. The prospective approach, e.g., the calib–zero [16] and empty–zero methods [17], estimates shading distortion from a set of image sequences for a reference slide obtained at various locations. Many commercial WSI devices provide the approach as a built-in function; however, acquiring image sequences of the reference slide whenever the brightness of light source changes requires additional effort, which is often prohibitive for many bioimaging researchers [15]. In contrast to the prospective approach, the retrospective approach estimates the shading distortion directly from actual image sequences that are acquired to generate the whole-slide image without the requirement of additional reference images.

The corrected intensity distributions using regularized energy minimization (CIDRE) [18] and background and shading correction (BaSiC) [15], which are categorized as the prospective approach, have been recently acknowledged as state-of-the-art methods, as they have achieved the best performances among 14 prospective and retrospective methods, as reported by reference [15]. The two methods assume smooth shading distortions. The CIDRE method estimates the shading distortion by minimizing a regularized energy function comprising a smoothness term, which enforces a smoothed shading distortion model. By contrast, the BaSiC method imposes sparse constraints on the Fourier-transformed shading distortion model to enforce smoothness. However, the two methods cannot accurately model the shading distortion of real images with fixed-pattern noise, which are a spatial variation in pixel values under uniform illumination [19], as the assumption suppresses spatial variations when estimating the shading distortion. The fixed-pattern noises should be corrected, as they resulted in the image quality degradation [20], which is especially visible when the high-resolution whole-slide images are zoomed in on the monitor screen.

We herein propose a shading correction method, which is straightforward, but robust against not only typical image artifacts caused by dust, scratches and bubbles, but also fixed-pattern noise that degrades the image quality. The proposed method comprises two steps. The first step reconstructs candidates for the shading distortion model by sorting intensities at each pixel axis from a stack of actual image sequences, and the second step selects the optimal shading distortion model, which does not include the typical image artifact, from the candidates by measuring the degree of smoothness. The proposed method removes the fixed-pattern noises in the process of correcting shading distortion, as the estimated flat-field distortion model contained information on the fixed-pattern noises. Therefore, the proposed method does not perform additional noise removal methods. We compared the proposed method with the two state-of-the-art methods, CIDRE [18] and BaSiC [15], for a quantitative evaluation. In the experiments, the proposed method showed better correction scores on diverse imaging conditions with fixed-pattern noise, as we did not impose additional smooth operations and constraints.

## 2. Experimental Setup

**Figure 1:**
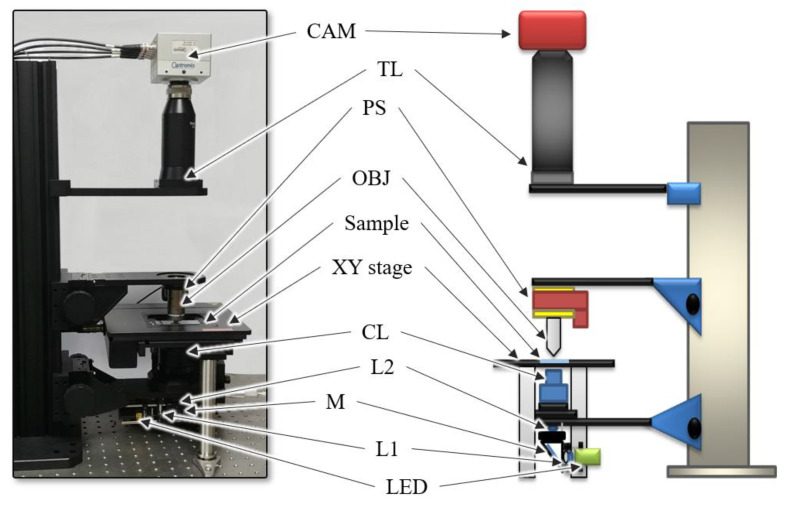
Schematics of the experimental setup. Light-emitting diode (LED): light source, L: lens, M: mirror, CL: condenser lens, OBJ: objective lens, PS: piezo stage, TL: tube lens and CAM: camera.

The schematics of the setup for the self-developed WSI device is shown in Figure 1. The device comprised four modules: a Köhler illumination to project brightfield light onto the sample, a motorized XY stage to move the sample along the x and y axes, a piezo stage to adjust the focus of the objective lens and a camera module to acquire sample images. We established Köhler illumination using a light source, two lenses (L1 and L2) and a condenser lens (CL) to generate an even illumination and ensure that the light source was not visible in the acquired images. The light source was a white light-emitting diode (LED) (MNWHL4, Thorlabs, Newton, NJ, USA). A 40 × 0.75NA objective lens (MRH00401, Nikon, Tokyo, Japan) and a tube lens (TTL200, Thorlabs, Newton, NJ, USA) were located between the condenser lens and the camera to project a magnified image of the sample on the sensor plane of the camera.

The image of the sample was acquired using a camera (CP80-4-C500, Optronis, Kehl, Germany) with a pixel size of 7 μm. The piezo stage (PD72Z4CAQ, Physik Instrumente (PI), Karlsruhe, Germany), which adjusts the focal plane of the objective lens, was used for the camera to acquire the focused image; the motorized XY stage (SCAN 75 × 50, Märzhäuser Wetzlar, Germany), which moves the slide along the x and y axes, was used to scan the whole slide. The image size of the camera used in the experiments was 2304 × 1720 pixels, and the FOV was 400 µm × 300 μm. The camera, motorized XY stage, and piezo stage were controlled by a self-developed software to generate digital slide images automatically, and the software was executed on an Intel Core i7-4790 CPU with 32 GB of memory.

## 3. Methods

The image formation process, which relates an image sequence $Y=\left[Y_{1},\ldots ,Y_{n}\right]$ acquired from the device and its corrected image sequence $X=\left[X_{1},\ldots ,X_{n}\right]$, can be approximated as a linear function [21], as follows:

(1) $$Y_{i}\left(k\right)=X_{i}\left(k\right)\times F\left(k\right)+D\left(k\right),$$

where F(k) denotes a flat-field distortion at the kth pixel caused by uneven illumination [17,22]; D(k) denotes dark-field distortion, which occurs if no light is incident [23]. Because the dark-field distortion is typically nearly uniform, varying by only a few intensity values [18], the proposed method assumes D(k)=0.

Therefore, the corrected image can be easily calculated by reversing the image formation process (Equation (2)) as follows:

(2) $${\hat{X}}_{i}\left(k\right)=\frac{Y_{i}\left(k\right)}{\hat{F}\left(k\right)},$$

where $\hat{F}\left(k\right)$ denotes the estimate of the actual flat-field distortion F(k). Because the actual distortion could not be determined precisely in practice, we estimated the flat-field distortion $\hat{F}$, in which the corrected image ${\hat{X}}_{i}$ optimally estimated $X_{i}$ from $Y_{i}$ [15,18,21].

**Figure 2:**
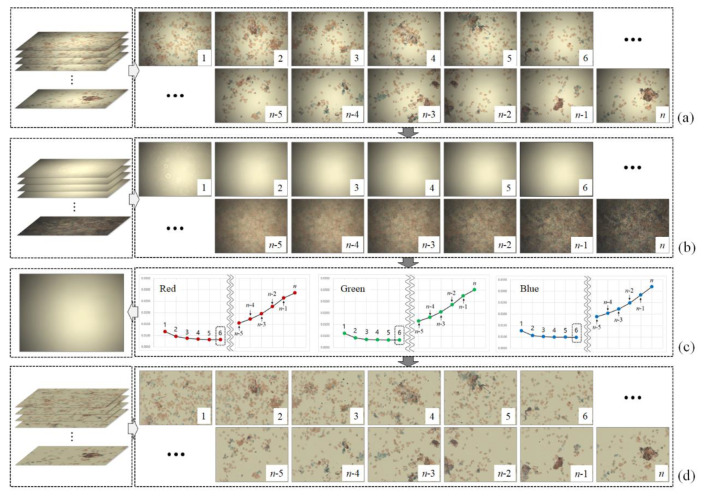
Flow of the proposed method: (**a**) input image sequence, (**b**) candidates of flat-field distortion, (**c**) selected flat-field distortion and (**d**) corrected image sequences.

Figure 2. shows the flow of the proposed method that estimates the flat-field distortion and corrects the shading distortion. The proposed method comprises primarily of two main steps. The first step constructs candidates for flat-field distortion (Figure 2b) from a stack of input sequences (Figure 2a). Using the brightfield WSI device allows the background pixels, which does not contain objects such as cells to block the illumination light, to have higher values than the object pixels. Based on this assumption, the proposed method constructs candidates by separately sorting the intensity values of red, blue and green channels at each pixel axis and then merging those three channels into a single RGB image. Figure 2b shows the candidates constructed from the image sequences in Figure 2a. The highest intensity values of three channels (the brightest pixel values) at each pixel axis are placed at the first candidate, whereas the darkest pixel values are placed at the last (*n*^th^) candidate.

**Figure 3:**
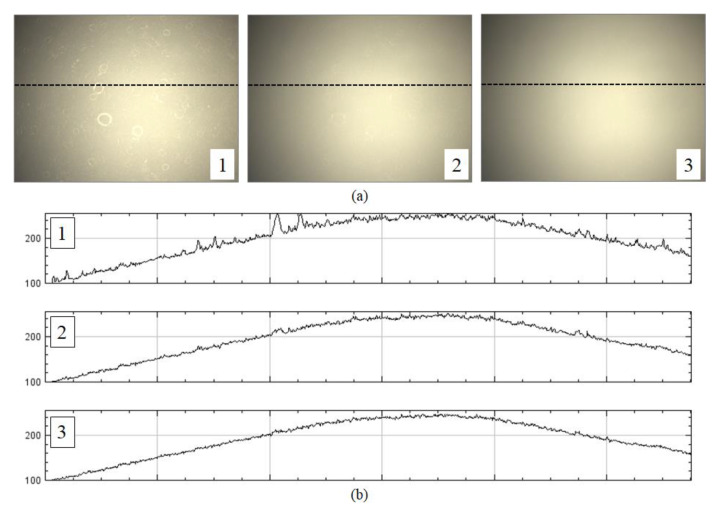
Example of artifacts reflected on candidates constructed with higher values: (**a**) enlarged images for 1, 2 and 3 of Figure 2**,** and (**b**) intensity profiles for a red channel on a dotted line.

In the second step of the proposed method, the optimal flat-field distortion among the candidates is selected. The candidate constructed with the highest values at each pixel axis can be considered as the optimal flat-field distortion. However, if typical artifacts, such as specks of dust, scratches and bubbles appear on the microscopic slide, they are reflected in the candidates constructed with higher values, as shown in Figure 3. Figure 3a shows more detailed (enlarged) images for 1, 2 and 3 of Figure 2b, while Figure 3b shows the intensity profile for a red channel on a dotted line segment. Therefore, estimating an optimal flat-field distortion robust to typical image artifacts can be considered as a problem of selecting a candidate with minimum artifacts. After estimating the flat-field distortion $\hat{F}$, input image sequences are corrected using Equation (2). Figure 2d shows result image sequences that the input image sequences (Figure 2a) are corrected by the estimated flat-field distortion (Figure 2c).

Candidates with more artifacts generally have more high-frequency components than those with no artifacts, as shown in Figure 3. Hence, the proposed method measures the degree of smoothness for each candidate. We define the degree of smoothness as the sum of the local coefficient of variation (LCoV), as the LCoV is used to determine whether a given pixel belongs to a high-frequency region or a homogenous region [24,25], as follows:

(3) $$S_{R}^{i}=\underset{k}{\sum }\frac{\sigma_{R}^{i}\left(k\right)}{\mu_{R}^{i}\left(k\right)},$$

where $\sigma_{R}^{i}\left(k\right)$ and $\mu_{R}^{i}\left(k\right)$ denote the local standard deviation and mean for a red channel at the kth pixel, respectively. A sliding window is used to compute the local standard deviation and the mean and the user determines the size of the sliding window. In the experiments, we set the size of the sliding window to 5 × 5 pixels. R in Equation (3) represents a red channel. The proposed method separately selects the optimal flat-field distortion model for each channel and then merges the distortion model for the abovementioned three channels into a single RGB distortion model. Figure 2c shows a graph (right of Figure 2c) representing the degree of smoothness of the candidates (Figure 2b) for each channel and the color shading model (left of Figure 2c) for the input images (Figure 2a).

## 4. Results

**Figure 4:**
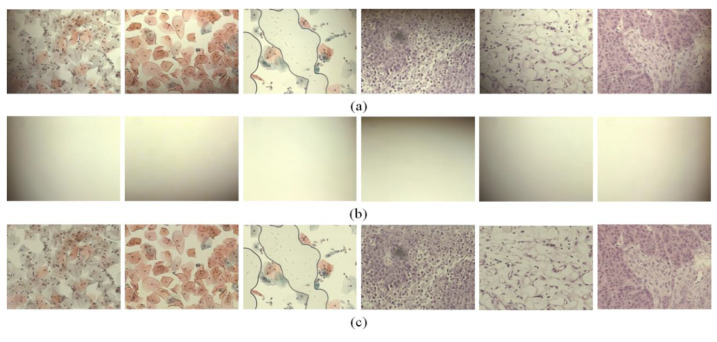
Examples of image tiles with shading corrected by the proposed method: (**a**) input image tiles, (**b**) flat-field distortion models and (**c**) shading-corrected images.

**Figure 5:**
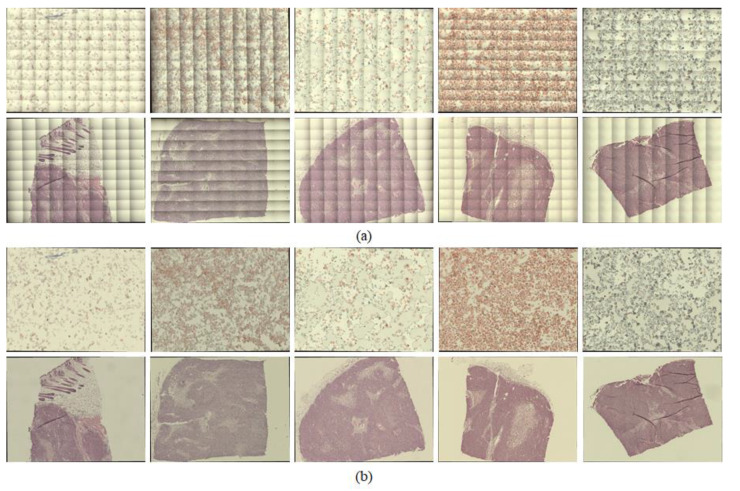
Examples of whole-slide images, with shading corrected by the proposed method: (**a**) whole-slide images before applying shading correction and (**b**) whole-slide images after applying shading correction.

In the experiments, we scanned various samples with different staining qualities and under varying imaging conditions using a self-developed WSI device to confirm the feasibility of the proposed shading correction method Figure 4. shows examples of images before (Figure 4a) and after (Figure 4c) applying the shading correction for each image tile. Here, the image tile is one of the image sequences acquired by the camera while translating the motorized XY stage to cover the whole slide. Figure 4a shows the image tiles for various samples. Figure 4b shows the flat-field distortion models estimated by the proposed method, whereas Figure 4c shows the result images corrected by each distortion model. Each sample used in the experiments exhibited different cell types and densities as well as different flat-field distortions. Figure 5 shows examples of the shading correction results for the whole-slide images; Figure 5a,b show the whole-slide images before and after applying the shading correction, respectively. We used the microscopy image stitching tool available in ImageJ [26], which stitches partially overlapped image sequences, to generate whole-slide images.

**Figure 6:**
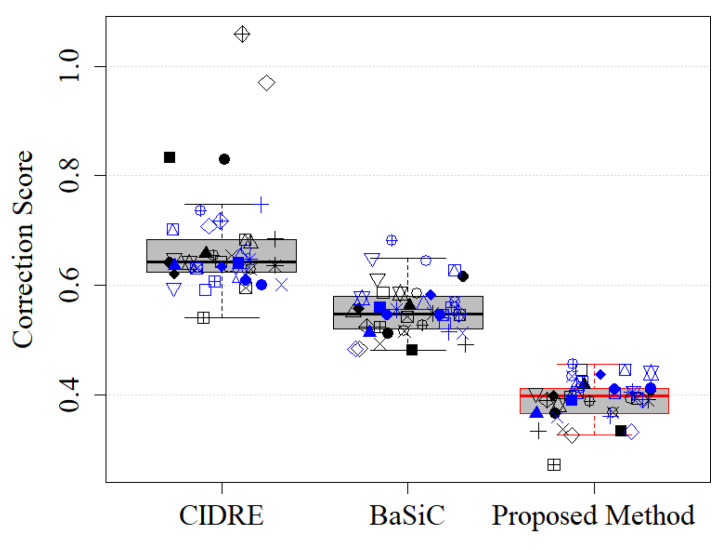
Correction scores of regularized energy minimization (CIDRE), BaSiC and the proposed method. Each symbol represents one of 40 image collections.

The proposed method was compared with two state-of-the-art methods, CIDRE [18] and BaSiC [15], for a quantitative evaluation. To assess the quality of the correction, we quantified the error of the shading-corrected image ${\hat{X}}_{i}$ with a correction score [15] Γ as follows:

(4) $$\Gamma =\frac{\sum_{i}\left|{\hat{X}}_{i}-X_{i}^{\mathrm{true}}\right|}{\sum_{i}\left|Y_{i}-X_{i}^{\mathrm{true}}\right|},$$

where $X_{i}^{\mathrm{true}}$ denotes the ground-truth image. The ground-truth images in the experiments were obtained based on the empty-zero method [17], which estimated the shading distortion as the average of images of an empty slide obtained at various locations. The correction score Γ is in the interval [0,∞), where 0 indicates perfect correction, 1 indicates the same amount of error as the uncorrected image and >1 indicates a greater disagreement. Figure 6 shows the correction scores of the proposed method and the two previous methods, CIDRE and BaSiC. The correction scores, averaged over 40 image collections, are as follows: the proposed method, 0.39 ± 0.099; the CIDRE method, 0.67 ± 0.047; the BaSiC method, 0.55 ± 0.038. Each symbol in Figure 6 indicates one of 40 image collections and each image collection consists of 100 images.

**Figure 7:**
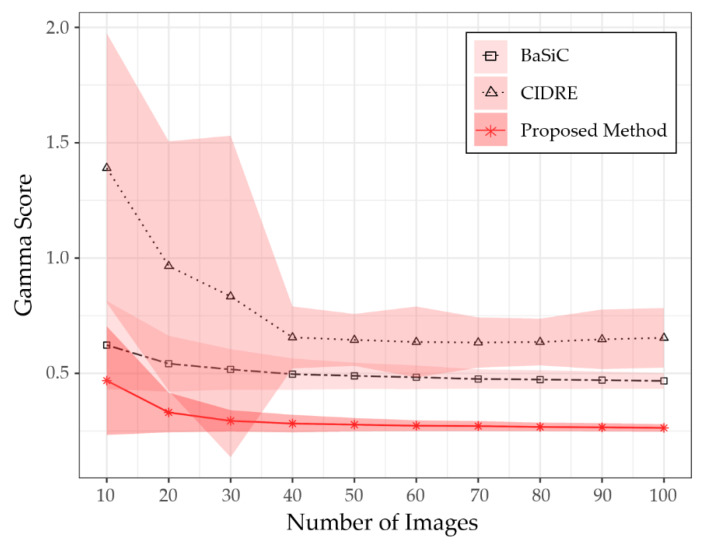
Comparison of gamma scores for number of input images.

To verify the minimum number of images to obtain the correction score given in Figure 6, we evaluated the performance of the proposed method as a function of the number of images. Figure 7 shows a graph representing correction scores according to the number of images used to model the shading distortion. The *x*-axis of the graph represents the number of images, randomly selected from each collection. The *y*-axis represents the correction scores, averaged over five different sets of images randomly selected from each component. In Figure 7, each curve is the average score of the five different sets of images, while the shaded area represents the standard deviation. As shown in Figure 7, the proposed method obtained the correction score given in Figure 6 if more than 30 images are given to model the shading distortion.

**Figure 8:**
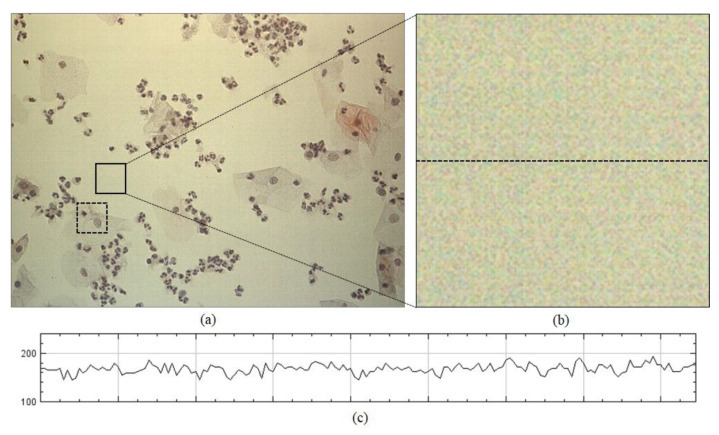
Example of fixed-pattern noise: (**a**) input image tile, (**b**) enlarged image and (**c**) intensity profile for the red channel on dotted line segment of (**b**).

**Figure 9:**
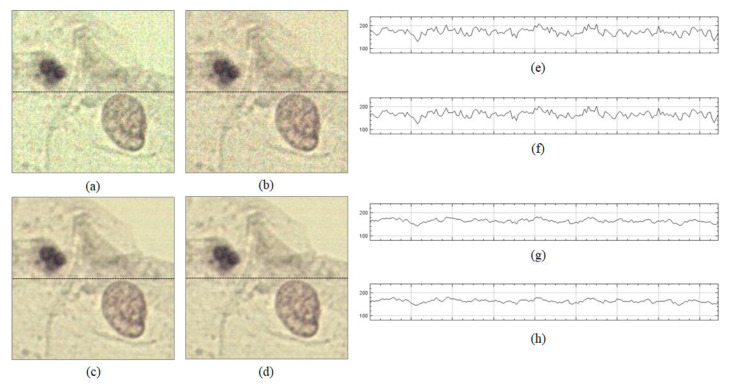
Shading correction results of two previous methods and the proposed method: (**a**) result image corrected by CIDRE method, (**b**) result image corrected by BaSiC method, (**c**) result image corrected by the proposed method, (**d**) ground-truth image, (**e**) intensity profile on dotted line of (**a**), (**f**) intensity profile on dotted line of (**b**), (**g**) intensity profile on dotted line of (**c**) and (**h**) intensity profile for the red channel on dotted line of (**d**).

The proposed method outperformed the two previous state-of-the-art methods because it could estimate the flat-field distortion that was robust to the fixed-pattern noise. The statistical test was performed for comparison between the means using the Wilcoxon signed-rank test, used in BaSiC [15]. It showed a significant difference between the proposed method and the two methods (n = 100, *p*-value = 1.819 × 10^−12^). Figure 8 shows an example of the fixed-pattern noise, which is a spatial variation in pixel values under uniform illumination, where Figure 8b shows an image in which a specific area of the input image is enlarged, whereas Figure 8c c shows the intensity profile for the red channel on the dotted line segment of Figure 8b. Figure 9 shows the resulting images that were shading-corrected by the two previous methods and the proposed method for the dotted box in Figure 8a. As shown in Figure 9, the results of the shading correction by the two previous methods still exhibited fixed-pattern noise, whereas it was reduced by the proposed method.

**Figure 10:**
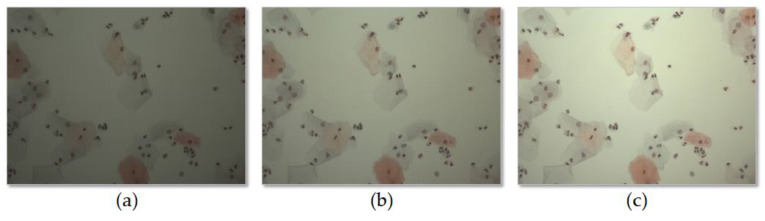
Example of image acquired at each brightness level. (**a**) 100, (**b**) 150, and (**c**) 200.

Since the shading distortion was greatly affected by the brightness of illumination, we evaluated the performance of shading correction according to the brightness of illumination. The brightness of the illumination was divided into three levels: 100, 150 and 200. Each level represents the average intensity of the images of an empty slide obtained at various locations. We acquired 40 image collections for each level, and the performance of the previous methods and proposed method was evaluated and compared for each level. Figure 10 shows an example of an image acquired at each level, and Table 1 shows the mean and variance of correction scores evaluated for each level. The experiments confirmed that the proposed method showed better correction scores than the previous methods regardless of the brightness of the illumination.

**Figure 11:**
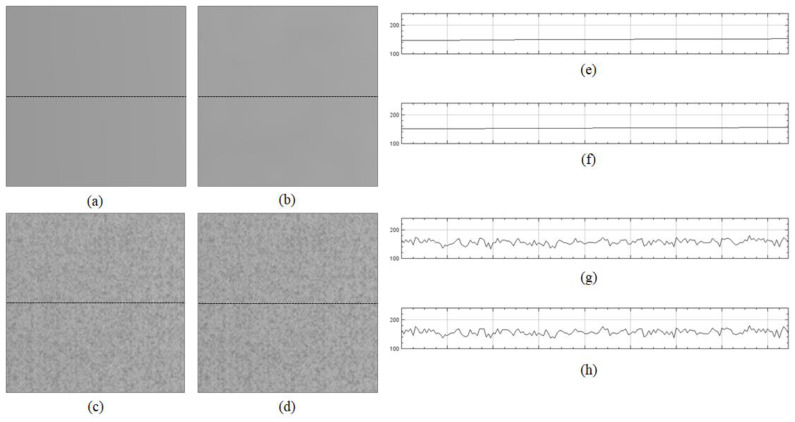
Flat-field distortion model estimated by two previous methods and the proposed method: (**a**) distortion model estimated by CIDRE method; (**b**) distortion model estimated by BaSiC method; (**c**) distortion model estimated by the proposed method; (**d**) ground-truth model; (**e**) intensity profile on dotted line of (**a**), (**f**) intensity profile on dotted line of (**b**), (**g**) intensity profile on dotted line of (**c**) and (**h**) intensity profile on dotted line of (**d**).

The fundamental difference between the previous methods and the proposed method is that the former assumes the smoothness of a flat-field distortion. The proposed method does not enforce the smoothness, which can eliminate information for fixed-pattern noise when estimating the flat-field distortion, whereas the previous methods assume smooth distortions. Figure 11 shows the flat-field distortion models estimated by the two previous methods (Figure 11a,b) and the proposed method (Figure 11c). The result images in Figure 1 were generated by the distortion models shown in Figure 11. As shown in Figure 11a,b,e,f, the flat-field distortion models of the two previous methods were smooth, which caused fixed-pattern noise to appear in the shading-corrected images of Figure 9a,b, as the input image sequences included the fixed-pattern noise, as shown in Figure 8. Meanwhile, the proposed method reduced the fixed-pattern noise as the flat-field distortion model estimated by the proposed method contained the necessary information, as shown in Figure 11c,g. Therefore, the proposed method can reduce fixed-pattern noise compared with the two previous state-of-the-art methods.

**Figure 12:**
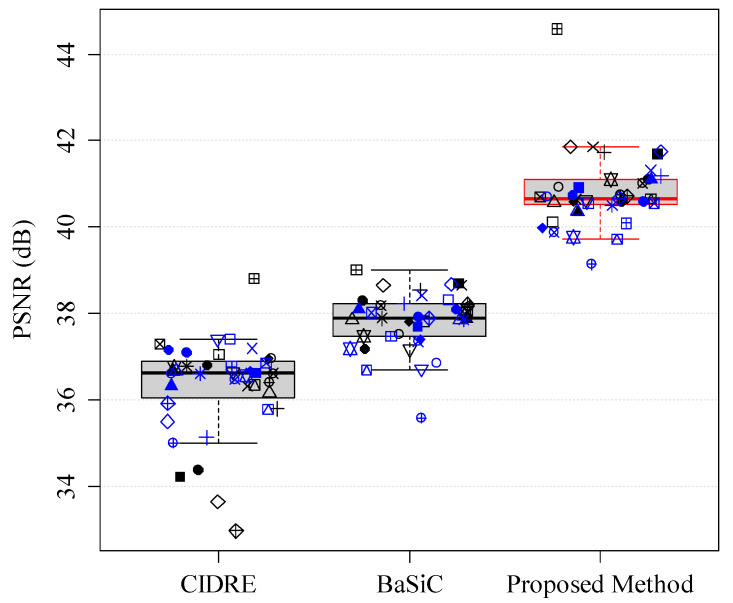
Peak signal-to-noise ratio (PSNR) of CIDRE, BaSiC and the proposed method.

Because the images that were shading corrected by the previous methods (Figure 9a,b) resembled those corrupted by noise, we additionally evaluated the quality of the shading-corrected images using a peak signal-to-ratio (PSNR). Figure 2 shows the PSNRs of the proposed and two previous methods. The higher the PSNR, the better is the quality of the shading-corrected image. The PSNRs, averaged over 40 image collections, are as follows: the proposed method, 40.80 ± 0.85 dB; the CIDRE method, 36.32 ± 1.09 dB; the BaSiC method, 37.83 ± 0.66 dB, respectively. Based on the quantitative evaluations with the correction scores and PSNR, we can confirm that the proposed method can correct not only shading distortion, but also fixed-pattern noise, compared with the two previous methods, as shown in Figure 6 and Figure 12.

To confirm that the proposed method is simple, but robust compared with the previous two methods, we evaluated the proposed method and the two previous methods in terms of the processing time. Since the processing time depends on the two factors, the number of images and the image size, we measured the processing time according to the two factors. Table 2 shows the processing time according to the image size and the number of images. The BaSiC code and the CIDRE code, written in Matlab, were downloaded from their official GitHub website at https://github.com/marrlab/BaSiC and https://github.com/smithk/cidre, respectively. The proposed method was also implemented using Matlab to measure the processing time in the same condition. As shown in Table 2, the proposed method showed the faster processing time, even if the CIDRE was almost constant regardless of the image size and the number of input images, because the processing time of the proposed method increased slowly according to the number of images and image sizes.

## 5. Conclusions

A simple shading correction method for brightfield WSI proposed herein, which was robust to not only typical image artifacts caused by specks of dust and bubbles, but also to fixed-pattern noise, which were spatial variations in pixel values under uniform illumination. The proposed method constructed candidates for the shading distortion model by sorting intensities at each pixel axis from a stack of input image sequences. This was based on the assumption that the brightfield WSI allowed the background pixels—which did not contain objects such as cells to block the illumination light—to have higher values than the object pixels. Next, the optimal shading distortion model that did not include the typical image artifact was selected from among the candidates, based on the local coefficient of variance. The proposed method did not enforce the smoothness, which could eliminate fixed-pattern noise information when estimating the shading distortion, whereas the two previous state-of-the-art methods assumed smooth distortions. Experimentally, we achieved better correction scores compared with the two previous methods under diverse imaging conditions with fixed-pattern noise, as the proposed method could correct not only shading distortion, but also fixed-pattern noise. However, the proposed method could not correct the shading distortion of the image sequences acquired by darkfield microscopy or fluorescence microscopy. In future studies, we will modify the proposed method to be applicable to non-brightfield WSI.

We searched other benchmark datasets to evaluate the proposed method but have not found datasets suitable for brightfield whole slide imaging. Therefore, we opened the image collections used in the experiments to GitHub at https://github.com/pair-kopti/Shading-correction. We expect that the dataset will be used to evaluate the novel shading correction method for brightfield whole slide imaging.

## Figures and Tables

**Table 1:** Comparison of correction scores according to the brightness of the illumination.

Brightness of Illumination	CIDRE	BaSiC	Proposed Method
100	0.6907 ± 0.0479	0.5909 ± 0.0270	0.4261 ± 0.0098
150	0.6667 ± 0.1035	0.5322 ± 0.0332	0.3034 ± 0.0087
200	0.6843 ± 0.1834	0.5171 ± 0.0417	0.3619 ± 0.1194

**Table 2:** Processing time (s) according to image size and number of images.

Method		No. of Images	100	200	300	400
	Image Size					
CIDRE	576 × 430		191.66	113.86	90.56	153.11
	1152 × 860		205.4	98.82	120.62	154.97
	2304 × 1720		231.03	133.22	142.44	248.76
BaSiC	576 × 430		343.96	522.44	395.56	520.59
	1152 × 860		1446.13	2226.99	1629.79	2388.19
	2304 × 1720		3213.24	4765.28	3561.35	5320.58
Proposed Method	576 × 430		12.52	18.6	25.08	62.77
	1152 × 860		29.76	44.36	60.13	152.21
	2304 × 1720		99.27	150.51	201.99	524.71
